# Uses of selection strategies in both spectral and sample spaces for classifying hard and soft blueberry using near infrared data

**DOI:** 10.1038/s41598-018-25055-x

**Published:** 2018-04-27

**Authors:** Menghan Hu, Guangtao Zhai, Yu Zhao, Zhaodi Wang

**Affiliations:** 1 Shanghai Jiao Tong University, Institute of Image Communication and Information Processing, Shanghai, China; 2 Shanghai Jiao Tong University, Department of Biomedical Engineering, Shanghai, China

## Abstract

In the current work, we attempt to leverage the fewer wavelengths and samples to develop a classification model for classifying hard and soft blueberries using near infrared (NIR) data. To do this, random frog selection and active learning approaches are used in the spectral space and the sample queue, respectively. To reduce the spectral number, a random frog spectral selection approach was applied to collect wavelengths informative with hardness. Prediction model based on 22 selected spectra gave slightly better results than that based on the full spectra. In terms of the selection operation in the sample space, the query by committee was validated to be suitable for blueberry hardness classification with the accuracy, precision and recall of 78%, 74% and 98% when taking only 25 sample queries. Its standard deviation curves of performance metrics are also located in regions of low values (around 0.05) and fluctuated steadily in shape, winning over those of the other 4 active learning strategies and random method. In summary, the respective uses of random frog and query by committee in the NIR spectral vector and the sample queue showed the considerable potential for establishing a simple but robust classifier for hard and soft blueberries with very low labeling cost.

## Introduction

Blueberry (*Vaccinium corymbosum*) is widely recognized as one of the important small soft fruit because of its reported antioxidant capacity^1^ and abundant phenolic compounds^2^. In the course of handling and transportation, a lot of the force loading conditions such as impact and compression as well as vibration will be loaded on the berry fruit^3^, thus extremely likely leading to the mechanical damage of berry fruit^4^. Such mechanical damage may alter the fruit textural properties and even cause the biological contamination, which in turn reduces the income of the fruit industry and brings the possible hazard to consumers^5,6^. For blueberry industry, to avoid the amount of economic loss, diversified transportation and marketing strategies are often required. One possible effective strategy is to classify berries into the hard and soft categories, and we can formulate different postharvest treatments and distribution means for these two hardness groups. For example, the harder category can be packaged for long-distance transport, and the softer category for short-distance transport.

The subjective nonoral hardness measurement can be used to sort hard and soft berries. However, efficiency of the nonoral method will decrease by the fatigue resulted from long time operation^7^. To overcome the limitation of subjective methods, objective measurement techniques including destructive and non-destructive approaches have been developed. Among them, some electronic and mechanical systems are capable of determining the hardness of berry fruit^8,9^, but these measurements are labor-intensive, time-consuming, sample-selection examination^10^.

In comparison with the hand-picked method and the abovementioned instrumental measurement, optical non-destructive measurement techniques are attempted to examine the external or internal quality of blueberry. Li *et al*., distinguished different growth stages using the traditional RGB camera under natural outdoor illumination^11^. They achieved the classification accuracy of 90% for mature and near-mature berry fruit via the use of the newly developed ‘SK-means’ classifier. A similar imaging device was applied by Zhang *et al*., for detection of bare spots in wild blueberry fields^12^. Some groups of investigators used hyperspectral reflectance and transmittance imaging system to estimate blueberry mechanical properties, and acceptable results were obtained^13,14^. Hu *et al*., utilized the rarely used hyperspectral interactance imaging architecture to successfully evaluate blueberry textural indicators^10^. Apart from the internal quality measurement, some investigators tried to eliminate the damaged blueberries from the sound ones using non-destructive optical methods. Jiang *et al*., applied the near-infrared (NIR) hyperspectral reflectance imaging system for detecting and quantifying blueberry bruising^15^. Fan *et al*., fused visible and very near-infrared and NIR hyperspectral reflectance imaging systems together to inspect the bruise of blueberry^16^. Hu *et al*., established hyperspectral reflectance, transmittance and interactance imaging modes to recognize the damaged berries. The results demonstrated that the hyperspectral transmittance data in conjunction with five pattern recognition algorithms could respectively give satisfactory detection accuracies^4^.

Nonetheless, damage detection belongs to the post-screening operation. With respect to the fresh agricultural produce industry, the best way to reduce the economic loss is to prevent damage from its formation. Hence, for the blueberry industry, we are able to conduct the assessment of mechanical characteristics of blueberries before they get injured. Based on the assessment, the corresponding handling processing strategy is afterwards formulated to minimize the number of damaged blueberries. The former paragraph has briefly reviewed some research work that can be used for the proposed assessment. However, in practical applications, we are more concerned with the grades of these internal quality indicators. Therefore, in the current work, we will focus on solving the problem of blueberry hardness classification.

In addition, there exist limitations in previous research work regarding blueberry quality. One is that the developed models do not take biological variations such as seasonal and cultivar variations into account. In our previous publications, we had found that the obtained prediction models would be more robust with biological variations introduced^17^. Zhang *et al*., also elaborated influences of physical and biological variability on inspection accuracy of the final model^18^. The other is that the extensively used non-destructive optical detection technique viz. hyperspectral imaging contains a large number of data. This indicates that the hyperspectral imaging technique will not be widely used in practice within a short time. In practical applications, we can develop a multi-spectral imaging system from the hyperspectral imaging system via the use of some informative wavelengths selection algorithms. This operation can also reduce the complexity of model, and thus increase robustness. However, to guarantee good detection accuracy of the established models, the number of ultimate retained wavelengths is usually more than 20^4,10,14^ for both hardness prediction and damage detection.

Consequently, this study is going to leverage NIR data inclusive of biological variations and selection strategies in both spectral and sample spaces to classify hard and soft blueberries. With respect to the NIR applications for blueberry, Sinelli *et al*., validated that there were good correlations between chemical compounds of blueberry and NIR spectral signals using partial least squares (PLS) regression algorithms^19^. Beghi *et al*., proposed the simple fruit classification algorithm using Vis-NIR spectral data for blueberry ripeness grading^20^. Yang *et al*., conducted spectral signal analysis for the classification of blueberry fruit and its leaves^21^. Zhang *et al*., measured the optical properties of healthy and bruised blueberry using spectroscopy operating in the NIR spectral region. Their results indicated that the reduced scattering coefficient and scattering anisotropy could be used as the merit for differentiating bruise^22^.

In order to obtain a better and simpler as well as low-cost classification model for blueberry hardness, we applied the random frog variables selection and active learning algorithms to the spectral and sample spaces, respectively. Numerous variables selection algorithms had been used in the NIR data^23^. Unlike the other approaches, random frog is a state-of-art selection algorithm that was first applied for selecting cancer-related genes^24^, and widely adopted in agricultural engineering^25,26^ because of its excellent performance. Application of random frog algorithm can ensure the simpler model and hence avoid the overfitting problem. The active learning technique, a subfield of machine learning, is applied as a selection strategy in the sample space and aims to achieve good classification by applying labeled samples as few as possible^27^. This algorithm can intelligently choose a small and informative subset from the entire blueberry dataset during the learning process, thus acquiring efficient and accurate as well as robust classifiers with less training. To our knowledge, in the field of agricultural engineering, there is no related literature using two selection strategies for both the feature and sample vectors.

The objectives of current study are to: (1) establish blueberry NIR spectral database containing a total of 684 samples with biological variations; (2) implement the random frog algorithm to extract the spectra particularly related to the hardness and develop the prediction model of blueberry hardness using selected wavelengths; and (3) use a certain amount of informative samples selected by the active learning technique to obtain low-cost classifiers of blueberry hardness.

## Results and Discussion

### Distribution of blueberry hardness

**Figure 1:**
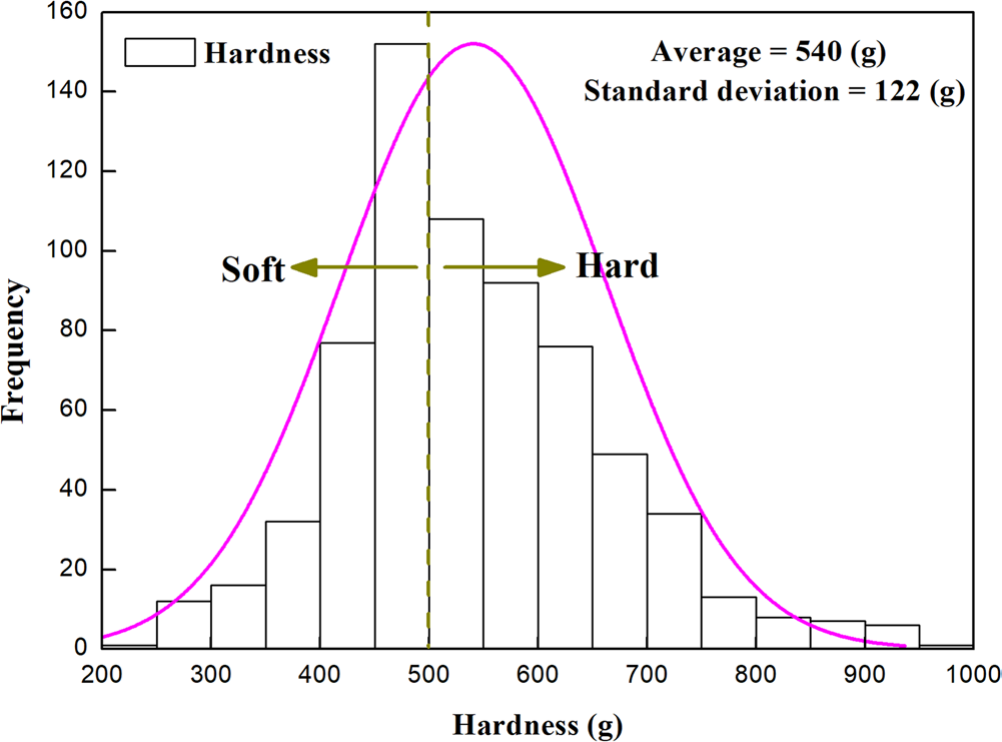
Distribution of hardness of blueberry.

The distribution of gathered hardness of blueberry is shown in Fig. 1. The hardness of 500 g is applied as the boundary to divide the samples into soft and hard berries (Fig. 1).

### Random frog for spectral space

**Figure 2:**
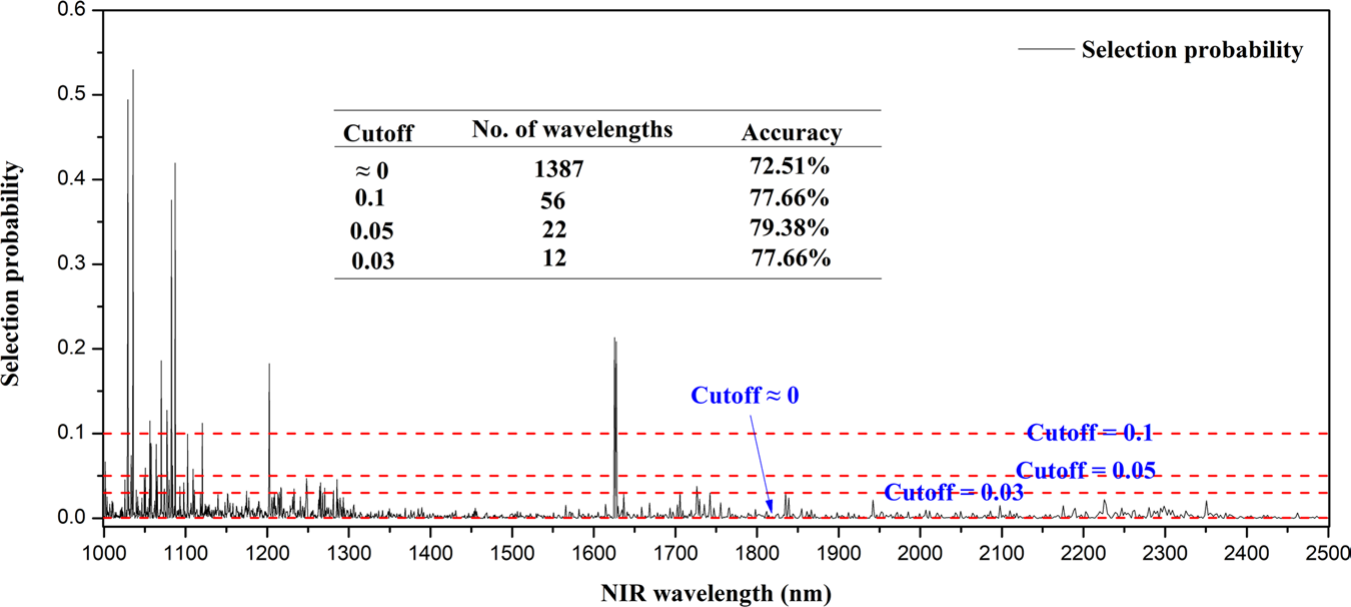
Selection probability of NIR wavelengths by random frog for blueberry hardness classification.

Figure 2 demonstrates the results of random frog for blueberry hardness using the parameter setting in Materials and Methods. As shown in Fig. 2, given the suitable cutoff threshold, the hardness-specific wavelengths can be extracted. To find this cutoff threshold, we compared the performances of 4 commonly used cutoff values. It can be seen in the sub-table in Fig. 2 that the 0.05 cutoff yields the best accuracy than the other 3 cutoff values with the appropriate wavelength number (here is 22). Therefore, wavelengths whose selection probabilities are beyond 0.05 are retained for the following analysis. These 22 informative wavelengths are 1627.58 nm, 1625.54 nm, 1202.57 nm, 1120.46 nm, 1108.95 nm, 1102.35 nm, 1087.10 nm, 1083.92 nm, 1082.56 nm, 1077.61 nm, 1077.16 nm, 1070.05 nm, 1063.90 nm, 1057.83 nm, 1057.40 nm, 1056.10 nm, 1050.54 nm, 1035.44 nm, 1033.37 nm, 1029.27 nm, 1028.86 nm, 1001.44 nm.

These selected wavelengths are first used for quantitatively analyzing blueberry hardness. The prediction model developed by the whole wavelengths can estimate the blueberry hardness with the Rp (Rc) of 0.9393 (0.8408) and RMSEp (RMSEc) of 54.46 g (65.28 g). By the use of the 22 selected wavelengths, the established model can estimate the blueberry hardness with the Rp (Rc) of 0.9419 (0.8453) and RMSEp (RMSEc) of 51.76 g (62.19 g), which is slightly superior to the entire wavelength model. This indicates the random frog algorithm can not only reduce model complexity, but also increase the model performance.

**Figure 3:**
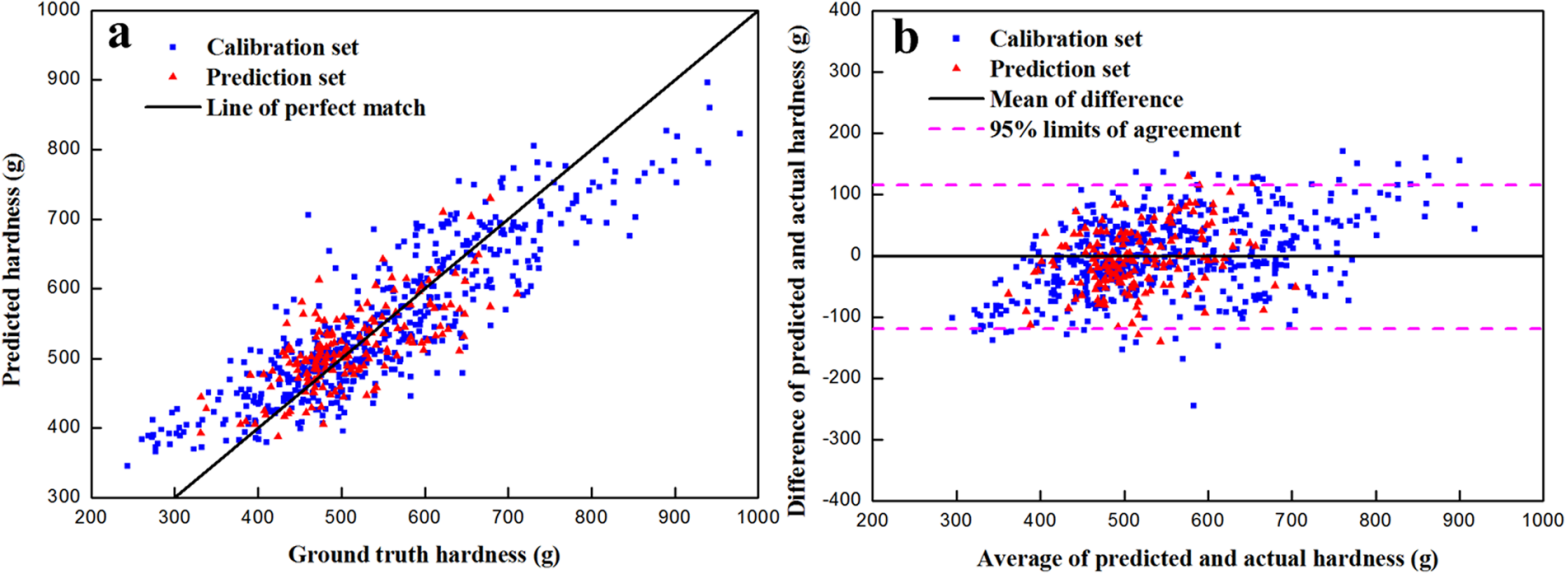
Graphical evaluation of performance of prediction model using random frog selected wavelengths: (**a**) linear correlation between ground truth and predicted hardness; and (**b**) Bland-Altman plot of the difference against average for predicted and actual hardness.

Figure 3 shows the results of graphical evaluation of prediction model using 22 random frog selected spectra. As shown in Fig. 3(a), most of the scatter points are close to the line of perfect match (slope = 1). By means of Bland-Altman analysis, we can observe that the majority of scatter points are dispersed around the mean of difference (here is −1.535 g) and within the 95% upper and lower limits of agreement (here is 115.627 g and −118.697 g, respectively) (Fig. 3(b)).

### Active learning for blueberry sample space

**Figure 4:**
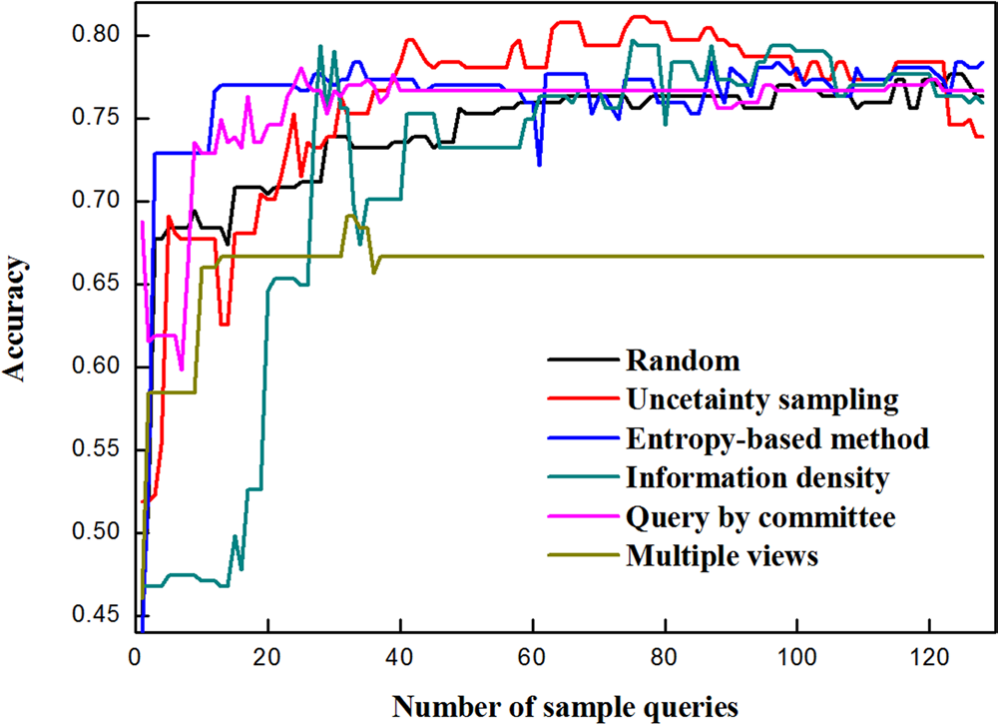
Accuracies of blueberry hardness classifiers modeled by active learning and random methods.

**Figure 5:**
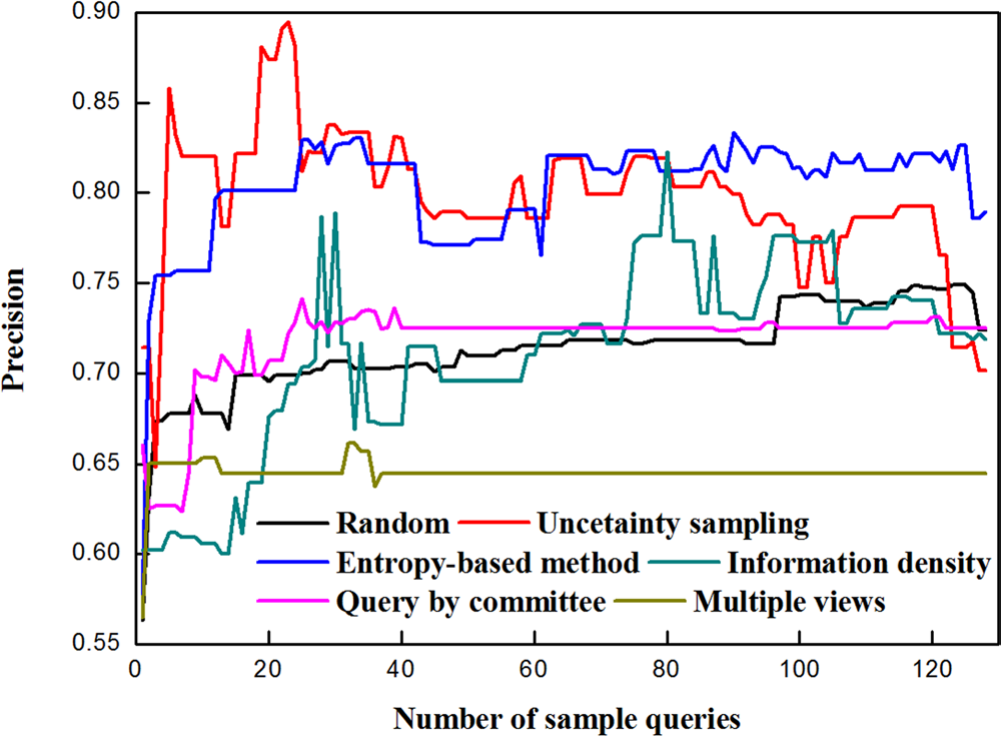
Precision of blueberry hardness classifiers modeled by active learning and random methods.

**Figure 6:**
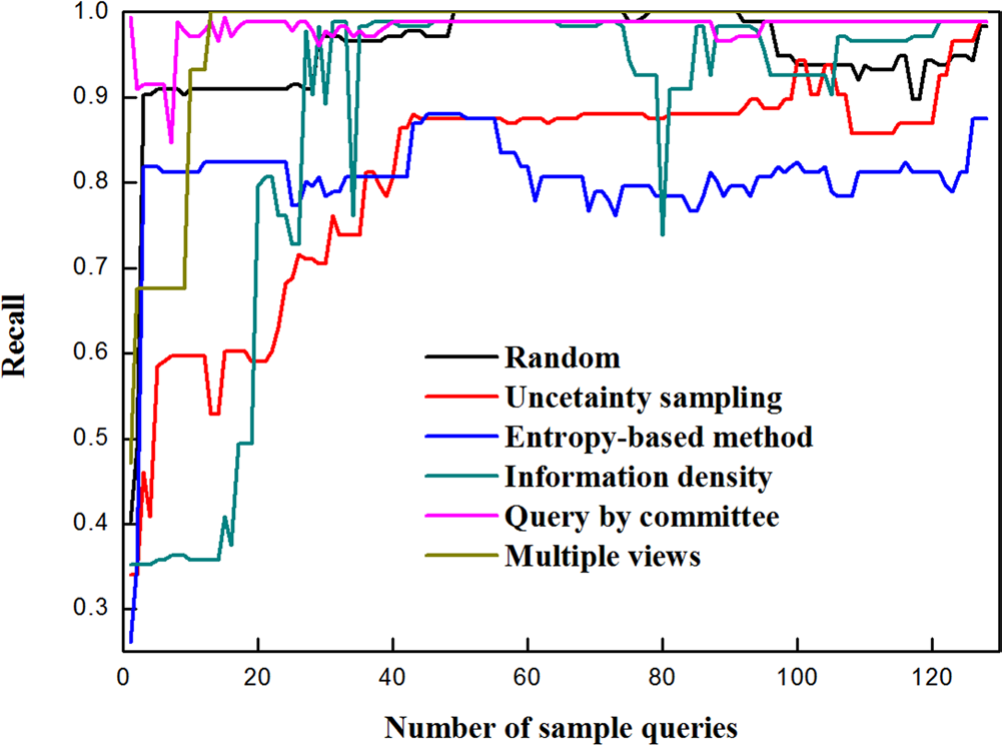
Recall of blueberry hardness classifiers modeled by active learning and random methods.

The above analysis has validated the effectiveness of random frog selected wavelengths for modeling the classifier of blueberry hardness. Subsequently, active learning algorithm was applied in the sample space to select the informative sample for modeling, and thus saved the labeling effort. The initial classifier was built by 4 randomly selected berries from the sample pool. In the current work, one active learning procedure finished after 128 sample queries. Each type of active learning and random method was run for 10 times. We picked the best results from 10 repeated runs via comprehensively examining the accuracy curve, and presented them in Figs 4–6.

Figure 4 exhibits the accuracy of classifiers for blueberry hardness classification using 5 active learning algorithms and traditional random sample selection. Apart from the multiple views, the other selection approaches produced comparable performances. The uncertainty sampling strategy achieved 80% accuracy at 40 queries, and maintained high accuracy for the remainder of the queries. The entropy-based method used 14 labeled samples to reach 77% accuracy, and the rest of queries also gave the accuracy around 77%. When 25 unlabeled samples had been queried, the query by committee could offer the accuracy of 78%, and the shape of the following curve is highly stable (around 77%). From Fig. 4, the performance of random method was better than the multiple views and information density.

Precision of blueberry hardness classifiers is shown in Fig. 5. For uncertainty sampling, its precision reached 86% very quickly after 5 queries were finished, and the best precision (90%) was appeared at 23 queries. However, the shape of uncertainty sampling’s curve fluctuated severely. The entropy-based method leveraged 14 queries to realize 80% precision, and the remaining queries could keep the precision around 80%. During query procedure, the precision of the query by committee was not high, but it was very stable (within 70% and 75%). The performance of random method was only better than that of multiple views.

Figure 6 shows the recall of blueberry hardness classifiers modeled by active learning and random methods. In terms of uncertainty sampling, the curve of recall was highly similar to that of accuracy in shape. Its recall came to 80% at 40 queries, and maintained over 80% until the end of queries. The entropy-based method could select 3 informative samples to make the recall curve rapidly increase to 90%. The excellent performance could be observed for the query by committee with nearly all recall rates beyond 90%.

From the above analysis, the uncertainty sampling and entropy-based method as well as query by committee outperformed the other methods, and could be applied for blueberry hardness classification to decrease the labeling cost.

**Figure 7:**
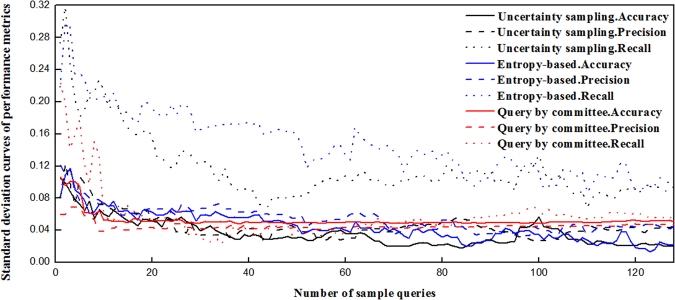
Standard deviation curves of 10 repeated runs for accuracy, precision and recall.

In order to evaluate the stability of these three active learning algorithms, we calculate the standard deviation curves of 10 repeated runs for accuracy, precision and recall. Figure 7 demonstrates the standard deviation curves. As shown in Fig. 7, the standard deviation curves of query by committee for accuracy, precision and recall located in the regions of low values (around 0.05) and change steadily in shape, winning over these of the uncertainty sampling and entropy-based method. Therefore, query by committee can be used to select informative berries in sample pool and lower the labeling cost. In addition, this active learning algorithm can avoid the uninformative, weakly informative, interference samples to affect the efficiency of model, which in turn optimizes the final model and its modeling processes.

## Conclusions

In conclusion, the random frog algorithm can select 22 hardness-specific wavelengths from raw NIR data pool. The hardness prediction model established by selected wavelengths outperforms the model established by the entire wavelengths with Rp (Rc) of 0.9419 (0.8453) and RMSEp (RMSEc) of 51.76 g (62.19 g) versus 0.9393 (0.8408) and 54.46 g (65.28 g), respectively. With respect to the selection strategy in the sample space, the performance metrics viz., accuracy, precision, and recall demonstrated that the query by committee, uncertainty sampling and entropy-based method gave the better overall performance for the classification of blueberry hardness than the random sample selection and the other active learning techniques viz., information density and multiple views. By examining the standard deviation curves, the uncertainty sampling and entropy-based method were not competitive. The classification model based on query by committee and 22 informative wavelengths can produce the accuracy, precision and recall of 78%, 74% and 98% with only 25 samples. Consequently, the combination use of random frog algorithm and query by committee can achieve the simple, stable and good classifier for blueberry hardness. This idea can be extended to other applications where the raw feature space is large and the labeled samples are difficult to obtain as well as the models are continually transferred.

## Materials and Methods

### Principle of used method

The principle of NIR spectrograph for classifying hard and soft blueberry is based on the fact that the different structures of berries (the macroscopic feature is the hardness) will alter the paths of incident light, thus changing the spectral patterns. Hence, the extraction and analysis of the obtained signals interacted with the blueberries can indirectly acquire their hardness information. Nonetheless, two main factors influence the final performance of the classification model. The first is that the NIR data are high-dimensional and co-linear, and the second is that the performance of classifier heavily depends on the number and quality of training dataset. In the current work, the random frog algorithm and active learning algorithm are used to address the co-linearity of NIR data and high labeling cost of training dataset.

### Blueberry Sample Preparation

Configurations of sample set are as follows: three blueberry cultivars viz. *Bluecrop*, *Duke* and *M2*, were grown in two producing areas viz. PR China and Chile and harvested during 2015 and 2016. A total of 684 blueberry samples at the commercial mature stage were purchased and transported to the lab located in Shanghai, PR China. All berries were stored at 4 °C during the experiments. To ensure the model robustness, only the blueberries without large visual injury were scanned by NIR spectroscopy and subjected to the subsequent invasive mechanical experiments.

### NIR data acquisition

**Figure 8:**
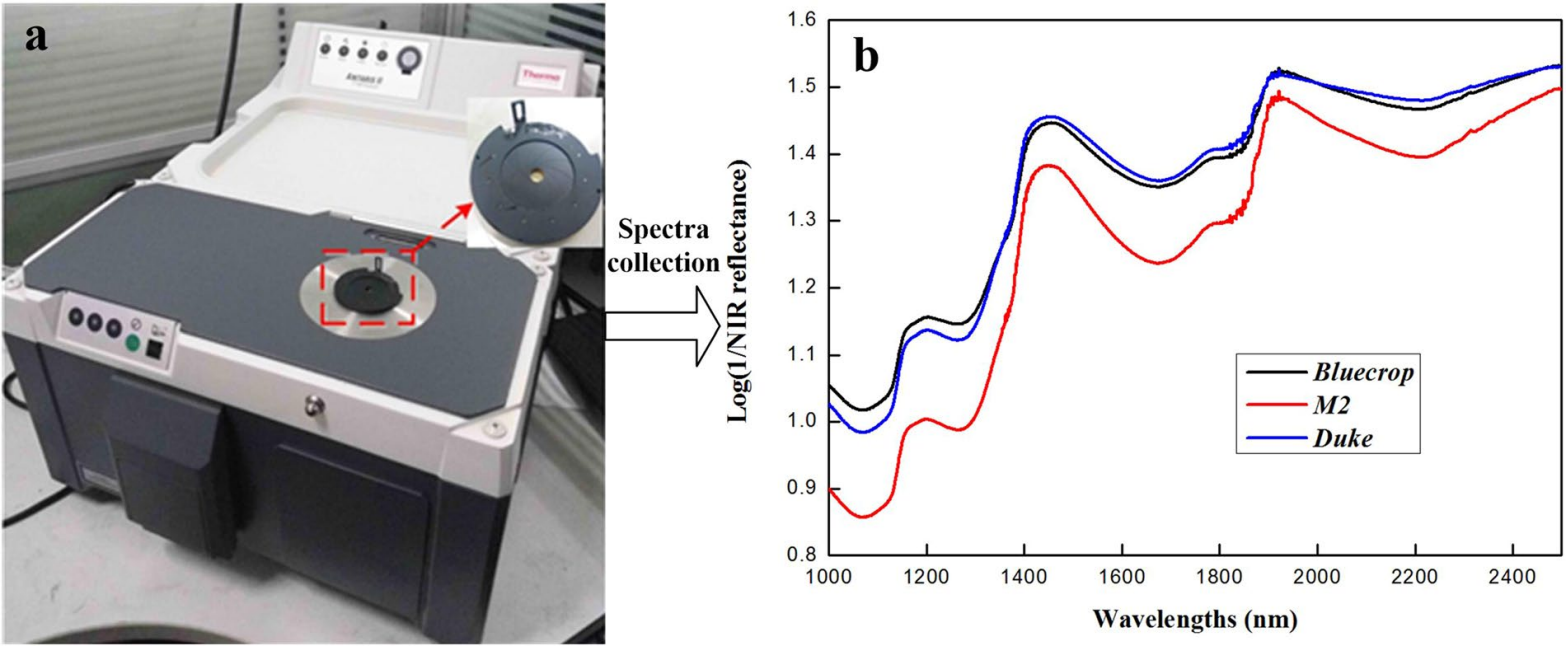
Photograph of the Antaris II FT-NIR spectrophotometer with the optical grating as the sample holder (**a**) and typical NIR spectral examples of three blueberry cultivars (**b**).

The FT-NIR spectra of intact blueberries were collected equatorially via the Antaris II FT-NIR spectrophotometer (Thermo Electron Co., USA) in reflectance mode with the integrating sphere. This NIR spectrophotometer was operated at the spectral range of 1000–2500 nm with the spectral resolution of 8.0 cm^−1^. Considering the features of blueberry sample, the gain of the spectrophotometer was fixed to 8 and each of the final spectrum was the average of 32 scans. Because the size of a blueberry is smaller than that of sample holder of Antaris II FT-NIR spectrophotometer for reflectance mode, the optical grating was placed on the original sample holder to hold the blueberry sample during spectra capture (Fig. 8(a)). The addition of this optical grating can prevent light leak and lower the impact of ambient light. The raw NIR spectra (R) were transformed into absorbance (log(1/R)) for the following data analysis. Typical spectral patterns of three blueberry cultivars are displayed in Fig. 8(b).

### Blueberry hardness measurement

**Figure 9:**
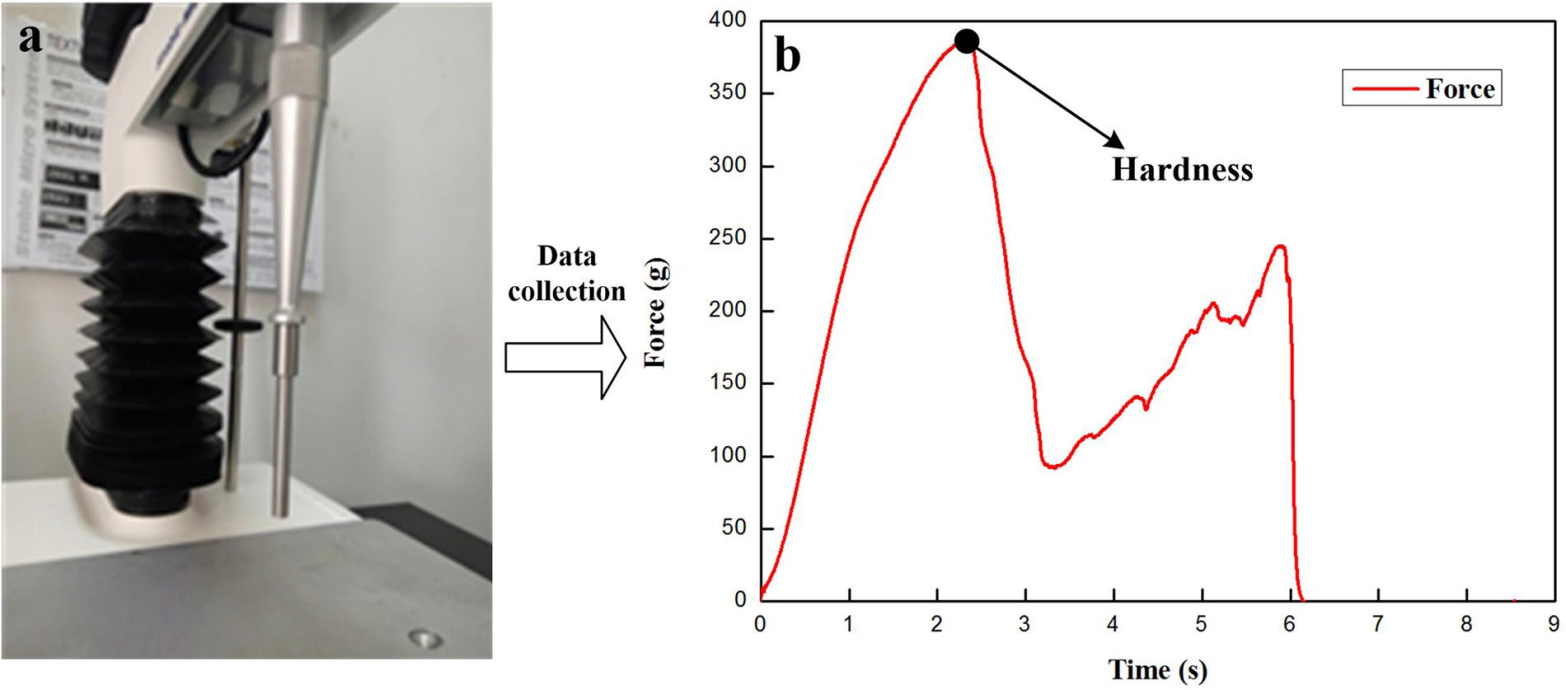
Photograph of puncture probe in 5 mm diameter (**a**) and typical force-time profile of blueberry derived from puncture analysis (**b**).

The hardness (g) of blueberry was measured by puncture analysis using a Texture Analyzer (TA.XTPlus; Stable Micro Systems, Godalming, UK). This instrument was equipped with the load cell of 50 kg and the puncture cylindrical probe of 5 mm diameter. The testing blueberry sample was placed on the cylindrical stainless flat platform with its stem scar facing vertically towards the probe. Figure 9(a) exhibits the experimental scenario. Before the puncture analysis, we should conduct the force and height calibration for the Texture Analyzer. The testing parameters of Texture Analyzer are as follows: auto force trigger = 5 g; digital data acquisition resolution = 500 point/s; deformation degree = 80%; test speed = 1.7 mm/s; pre-test speed = 2 mm/s; post-test speed = 5 mm/s. The hardness represents the maximum force during puncture, and it has been described in the force-time profile (Fig. 9(b)).

### Random frog for spectral space

The blueberry NIR spectral database was first divided into three parts using the improved Kennard-Stone sampling method^28^. A total of 100 berries were taken out for random frog analysis, and the remaining berries were used for the following active learning. For regression analysis, 75% and 25% of the rest of samples were selected as the calibration and prediction sets.

The steps of random frog proposed by Li *et al*.,^24^ are described as follows for seeking out the hardness-specific wavelengths: (1) configure the appropriate parameters: the numbers of latent variable and repeated run are set to 10 and 10,000, respectively. The selection probability and initial number of variable are fixed to 0.1 and 2, respectively; (2) select the candidate wavelengths: the normal distribution is used to form the original candidate wavelengths. During the repetition, the selection principle of the candidate wavelengths in current stage and in next stage can be referred to our previous study^14^; (3) determine the final candidate wavelengths: the selected frequencies of all NIR wavelengths are divided by the number of simulation (here is 10,000) to obtain the selection probability. Based on the principle of random frog, the larger selection probability suggests the more importance of the wavelength. Here, wavelengths whose selection probabilities are beyond 0.05 are taken as the resulting candidate wavelengths.

Prediction model of blueberry hardness was established using the Least Squares-Support Vector Machine (LS-SVM), and the performance of the final model was examined by the Pearson correlation coefficient of calibration (Rc) and prediction (Rp), root mean square error of calibration (RMSEc) and prediction (RMSEp). Also, the Bland-Altman plot was applied to check the effectiveness of the model^29^.

### Active learning for blueberry sample queue

In respect to the fresh agricultural produce industry, agronomists or agricultural engineers at present collect a variety of target samples and then annotate these samples using tedious, chemical-expensive, time-consuming and error-prone “gold standard” methods. Once these models are developed, they are very difficult to be updated or transferred to adapt other application scenarios or environments. In other words, we should redevelop the model for the new application purpose using the newly-collected labeled sample set.

**Figure 10:**
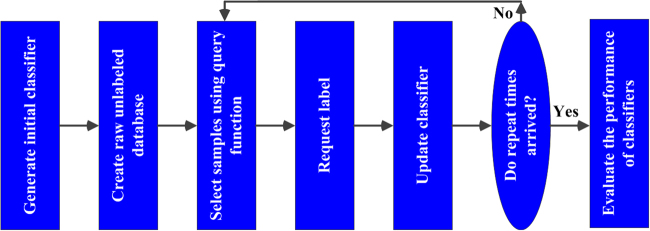
Procedure of active learning for blueberry hardness classification.

To avoid the above situations, we explore the feasibility of active learning algorithm for establishing a hardness classifier with very small sample size. A total of 5 active learning algorithms inclusive of 3 pool-based methods viz. uncertainty sampling^30^, entropy-based method^31^ and information density^32^, and 2 stream-based methods viz. query by committee^33^ and multiple views^34^ are used to rank the testing samples based on their informativeness. For comparison, the randomly ranked sample queue is also applied for modeling. Subsequently, the LIBSVM^35^ toolbox is adopted to classify hard and soft blueberries. The procedure of active learning for blueberry hardness classification is summarized in Fig. 10.

### Summary of two selection strategies for modeling

**Figure 11:**
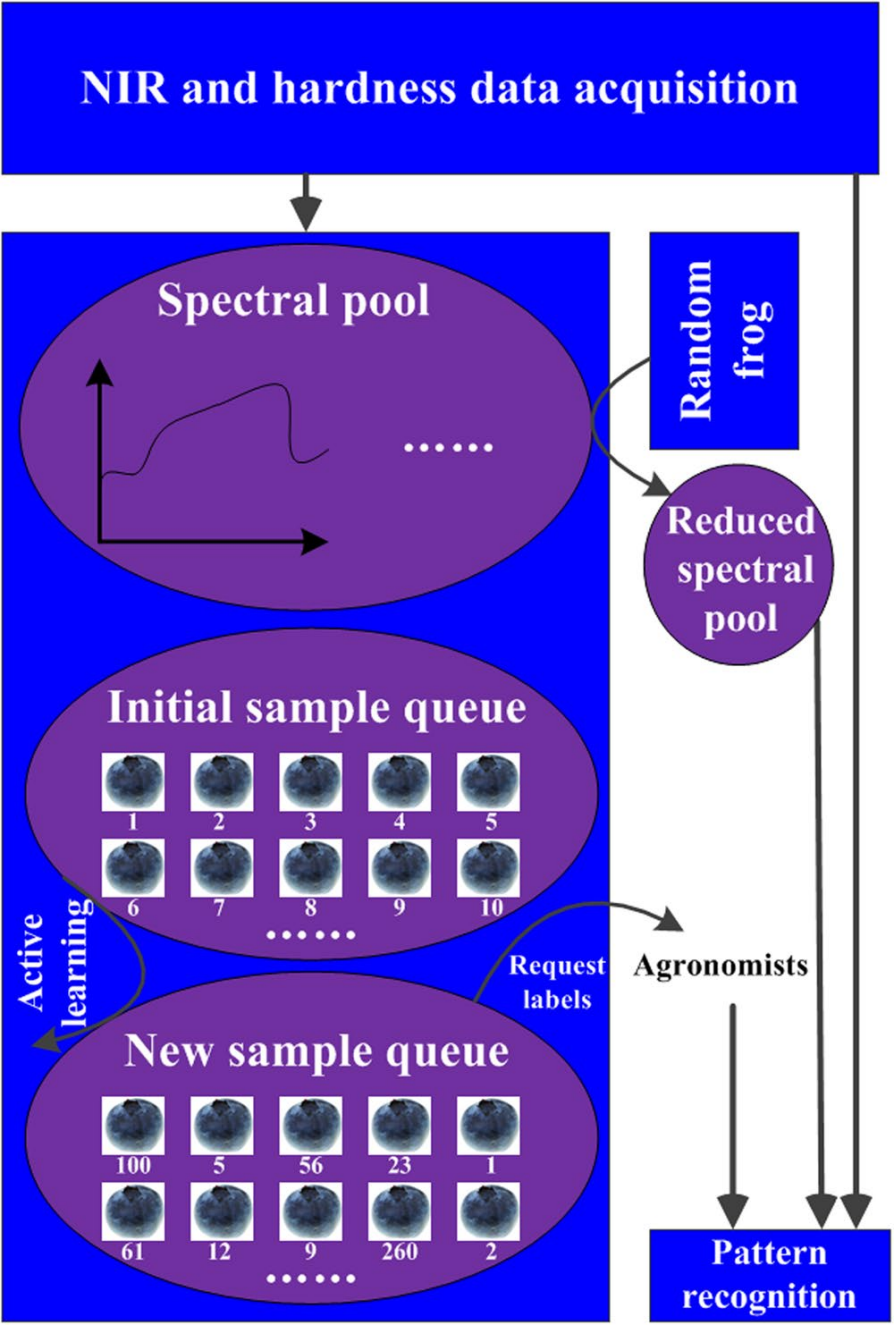
Procedure of two selection strategies in feature and sample spaces for blueberry hardness classification.

The entire procedure of two selection strategies for the classification of blueberry hardness is concluded in Fig. 11.

The effectiveness of established classifier was examined by three metrics namely accuracy, precision and recall. All the codes in this work were executed in the software of MATLAB R2014a (The Mathworks, Inc., Natick, Massachusetts).

## Electronic supplementary material

Dateset 1
